# EZH2 promotes metabolic reprogramming in glioblastomas through epigenetic repression of EAF2-HIF1α signaling

**DOI:** 10.18632/oncotarget.9761

**Published:** 2016-06-01

**Authors:** Bo Pang, Xiang-Rong Zheng, Jing-xia Tian, Tai-hong Gao, Guang-yan Gu, Rui Zhang, Yi-Bing Fu, Qi Pang, Xin-Gang Li, Qian Liu

**Affiliations:** ^1^ Department of Neurosurgery, Qilu Hospital of Shandong University, Jinan, 250012, Shandong, China; ^2^ Department of Neurosurgery, Shandong Provincial Hospital Affiliated to Shandong University, Jinan, 250021, Shandong, China; ^3^ Department of Gynecology and Obstetrics, Jinan Central Hospital affiliated to Shandong University, Jinan, 250013, Shandong, China; ^4^ Department of Histology and Embryology, Shandong University School of Medicine, Jinan, 250012, Shandong, China; ^5^ Department of Gynecology and Obstetrics, Shandong Provincial Hospital Affiliated to Shandong University, Jinan, 250021, Shandong, China

**Keywords:** EZH2, glioblastoma, Warburg effect, HIF1α, EAF2

## Abstract

Cancer cells prefer glycolysis for energy metabolism, even when there is sufficient oxygen to make it unnecessary. This is called the Warburg effect, and it promotes tumorigenesis and malignant progression. In this study, we demonstrated that EZH2, a multifaceted oncogenic protein involved in tumor proliferation, invasion and metastasis, promotes glioblastoma tumorigenesis and malignant progression through activation of the Warburg effect. We observed that HIF1α is a target of EZH2 whose activation is necessary for EZH2-mediated metabolic adaption, and that HIF1α is activated upon EZH2 overexpression. EZH2 suppressed expression of EAF2, which in turn upregulated HIF1α levels. We conclude from these results that EZH2 promotes tumorigenesis and malignant progression in part by activating glycolysis through an EAF2-HIF1α signaling axis.

## INTRODUCTION

Normal cellular energy metabolism differs from that in cancer cells. Cancer cells depend more on glycolysis than on mitochondrial oxygenic respiration, even when oxygen is sufficiently available (Warburg effect) [1, 2]. This characteristic is advantageous to cancer cells because it enables them to proliferate under hypoxic conditions, protects them from apoptosis, decrease their production of reactive oxygen species and enhance their drug resistance [3, 4]. In glioblastoma, glycolytic metabolism is about 3 times higher than in normal brain tissue and is regulated by some well-known oncogenes, including phosphoinositide 3-kinase, Akt and hypoxia-inducible factor 1 (HIF1) [5]. Although many of the genes contributing to the metabolic switch between the glycolytic and tricarboxylic acid (TCA) cycles are known [6], the metabolic pathways involved in glioblastoma cells are far from clear.

Enhancer of zeste homology 2 (EZH2) is the catalytic subunit of polycomb repressive complex 2, which represses gene expression by generating a methylated epigenetic mark at Lys27 of histone H3 (H3K27me3) [7, 8]. It appears that EZH2 is up-regulated in a variety of human malignancies, including breast, colorectal, prostate, cervical and lung cancers, as well as sarcoma and glioblastoma, where its expression is associated with cancer initiation, progression, metastasis and prognosis [9, 10]. Most of EZH2 target genes are tumor suppressors. For instance, INK4B-ARF-INK4A, p57, bone morphogenetic protein receptor 1B, MyoD and RUNX3 are all negatively regulated by EZH2, which is critical for tumor cell proliferation and aggressiveness [11–13]. E-cadherin gene (*CHD1*), another important target of EZH2, is involved in epithelial-mesenchymal transition (EMT), invasion and migration [14], while Bim, TRAIL and FBO32, three molecules involved in apoptosis [15, 16], and Vasohibin1, a molecule closely associated with tumor angiogenesis [17], are also suppressed by EZH2. But more than this, EZH2 appears to be a multifunctional protein in human malignancies. For example, it reportedly accumulates in the cytosol and mediates actin polymerization [18]. In addition, DNA double-strand break repair is prevented and tumor cells become more sensitive to ionizing radiation after EZH2 knockdown [19]. As a result of its having many actions, the precise mechanism by which EZH2 affects metabolic reprogramming, a hallmark of cancers, remains unclear.

ELL-associated factor 2 (EAF2) is a partner of eleven-nineteen lysine-rich leukemia (ELL). It was initially identified as a novel androgen-responsive gene in the prostate, where it is involved in the regulation of transcriptional elongation of RNA Poll II [20, 21]. Inactivation of EAF2 induces tumorigenesis in several organs, including lung, liver and prostate [22]. Although its mechanism of action is unknown, studies show that EAF2 functions in controlling the growth and survival of cancer cells by negatively regulating canonical Wnt/β-catenin signaling as well as the RAS-BRAF-ERK signaling pathway [23, 24]. EAF2 also reportedly binds to and stabilizes von Hippel-Lindau protein (pVHL), a tumor suppressor involved in mediating HIF1α degradation and an inhibitor of the hypoxia pathway, which suggests it is a potential metabolic regulator [25]. In this study, we demonstrated that EZH2 alters cellular metabolism in part through an EAF2-HIF1α signaling axis.

## RESULTS

### Overexpression of EZH2 in glioblastoma cells increases glycolytic metabolism

**Figure 1:**
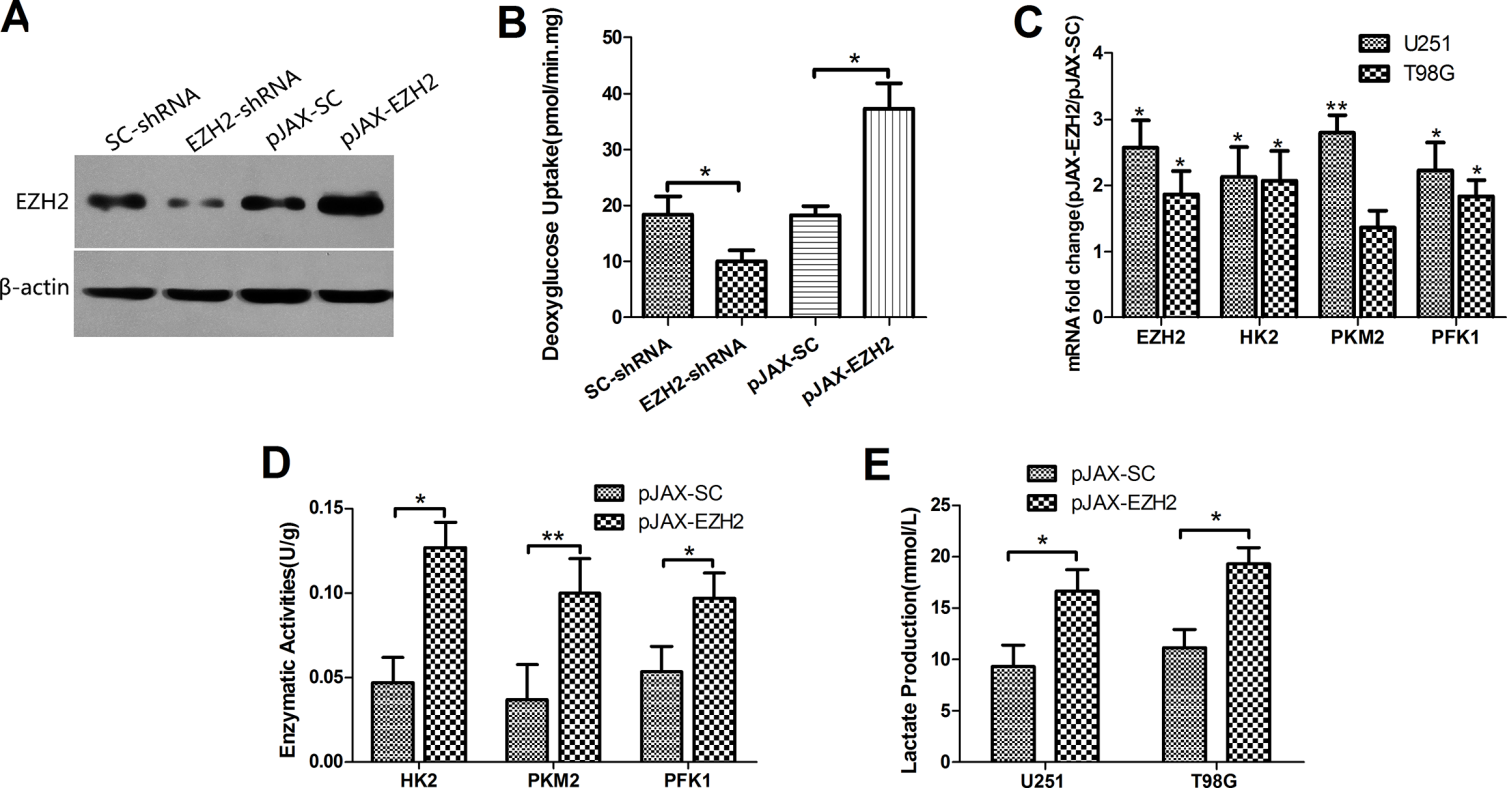
EZH2 is involved in the glycolytic metabolism in glioblastoma cells (**A**) Levels of EZH2 protein were analyzed by immunoblotting U251 glioblastoma cells transfected with SC-shRNA, EZH2-shRNA, pJAX-SC or pJAX-EZH2. β-actin served as a loading control. (**B**) Relative deoxyglucose uptake was measured in U251 cells transfected with SC-shRNA, EZH2-shRNA, pJAX-SC or pJAX-EZH2. Each bar represents the mean ± s.d. from three independent experiments. *P < 0.05. (**C**) mRNA levels of HK2, PKM2 and PFK1 were determined in the indicated cells. Each bar represents the mean ± s.d. from three independent experiments. *P < 0.05, **P < 0.01. (**D**) Enzymatic activities of HK2, PKM2 and PFK1 were determined in the indicated U251 cells. Each bar represents the mean ± s.d. from three independent experiments. *P < 0.05, **P < 0.01. (**E**) Lactate production was measured in U251 and T98G cells transduced with pJAX-SC or pJAX-EZH2. Data were presented as the mean ± s.d. from three independent experiments. *P < 0.05.

To investigate the effect of EZH2 on energetic transformation of glioblastomas, we separately introduced a pJAX vector encoding wild type EZH2 and a pSuper.neo vector encoding EZH2 shRNA into U251 (Figure 1A) and T98G (Supplementary Figure 1A) glioblastoma cells. The results indicated that EZH2 was substantially upregulated in the pJAX-EZH2 transfectants and depleted in the pSuper.neo-EZH2-shRNA transfectants.

To assess glycolytic activity, we measured cellular deoxyglucose uptake in U251 (Figure 1B) and T98G (Supplementary Figure 1B) cells. The results showed that up-regulation of EZH2 increased intracellular deoxyglucose levels. EZH2 knockdown, on the other hand, elicited the opposite effects.

We next assessed the mRNA expression and enzymatic activities of hexokinase (HK), pyruvate kinase (PK) and phosphofructokinase (PFK), three enzymes involved in the glycolytic pathway. The results showed that over-expression of EZH2 increased the mRNA expression (Figure 1C) and enzymatic activities of HK2, PKM2 and PFK1 in the U251 (Figure 1D) and T98G (Supplementary Figure 1C) cells.

Elevated lactic acid production is a feature of Warburg effect. Measurement of lactic acid levels in culture medium conditioned by U251 and T98G cells showed that overexpression of EZH2 led to increased secretion of lactic acid from the cells (Figure 1E).

### EZH2 promotes the switch from mitochondrial respiration to glycolysis

**Figure 2:**
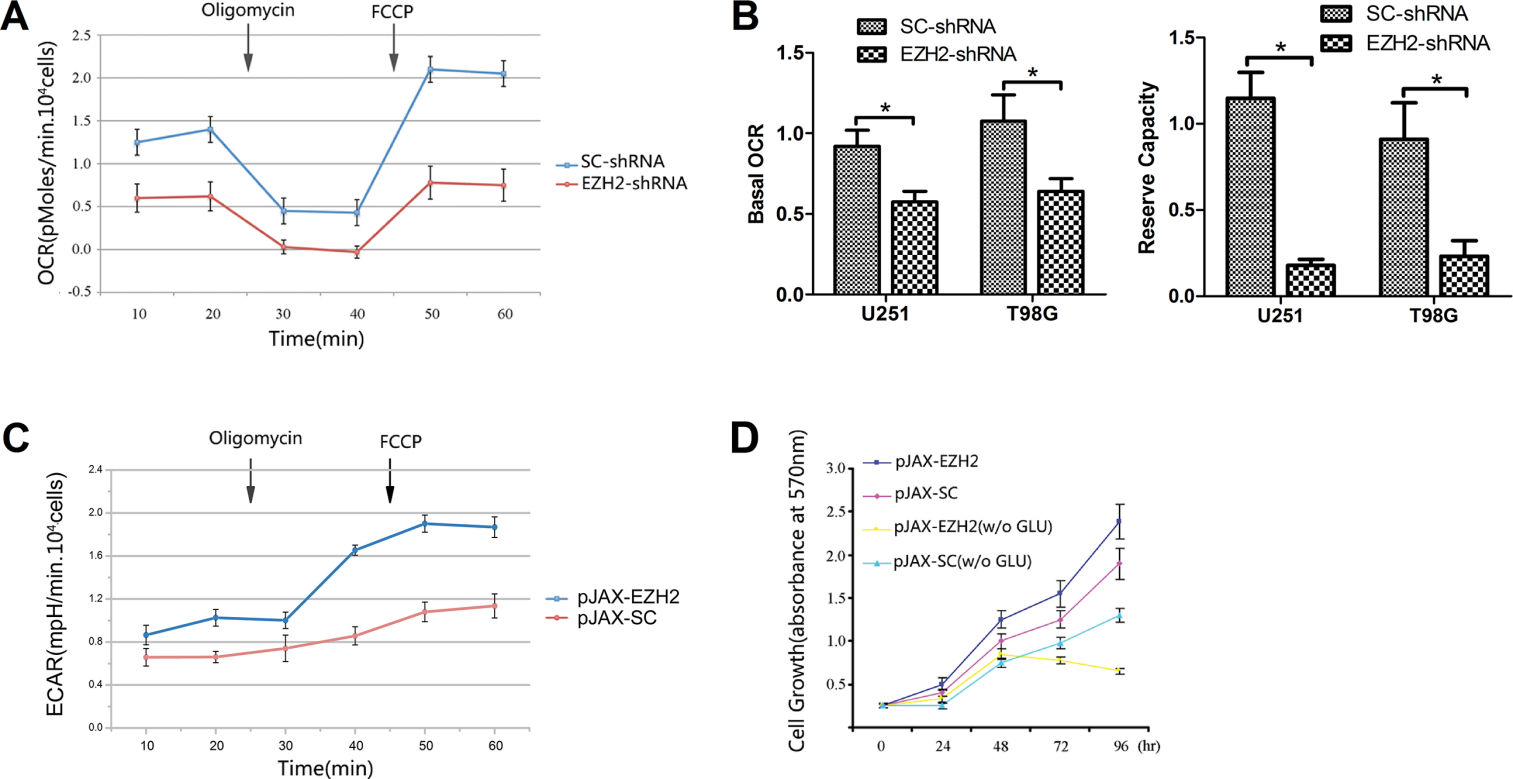
Upregulation of EZH2 induces an inclination toward glycolytic respiration (**A**) OCRs were determined using a Seahorse XF24 analyzer with U251 glioblastoma cells transfected with SC-shRNA or EZH2-shRNA. Oligomycin (1 mM) and FCCP (300 mM) were administered as indicated. (**B**) Basal OCR was calculated as the mean of original OCRs minus the mean of OCRs after addition of oligomycin. Reserve capacity was calculated as maximal OCR (FCCP-induced levels) minus basal OCR. Results are presented as the mean ± s.d. from three independent experiments. *P < 0.05. (**C**) ECARs were determined using a Seahorse XF24 analyzer with U251 glioblastoma cells transfected with pJAX-SC or pJAX-EZH2. Oligomycin (1 mM) and FCCP (300 mM) were administered as indicated. (**D**) Growth curves for the indicated U251 cells cultured in glucose-containing (4500 mg/l) or glucose-free (w/o GLU) medium. Each bar represents the mean ± s.d. from three independent experiments.

To examine the possible effect of EZH2 on mitochondrial activity in glioblastoma cells, we added oligomycin, pharmacological inhibitor of electron transport, and FCCP, a mitochondrial uncoupler, to transduced U251 (Figure 2A) and T98G (Supplementary Figure 1D) cells and then determined their oxygen consumption rates (OCRs) using an extracellular flux analyzer. The results showed that sequential addition of oligomycin and FCCP resulted in a decrease followed by an increase in the OCR in all transduced cells. Strikingly, EZH2 knockdown substantially reduced both the basal OCR and the calculated reserve capacity in U251 and T98G cells (Figure 2B), suggesting mitochondrial activity was reduced in these cells. Up-regulation of EZH2, on the other hand, elicited a minor increase in mitochondrial oxidative capacity (Supplementary Figure 1E). Nevertheless, the extracellular acidification rate (ECAR), estimated using a methodology similar to that used for OCR measurement, was higher and consistent with the lactate production data in Figure 1E (Figure 2C, Supplementary Figure 1F).

We next examined the growth kinetics of U251 cells with or without EZH2 over-expression. Up-regulation of EZH2 in the glioblastoma cells promoted cell proliferation. On the other hand, when cultured in glucose-free medium, growth of these cells was notably slower than control (Figure 2D), which implies that cells over-expressing EZH2 may depend on enhanced glycolysis as their major energy source.

### EZH2 is associated with metabolic reprogramming in intracranial glioma xenografts

**Figure 3:**
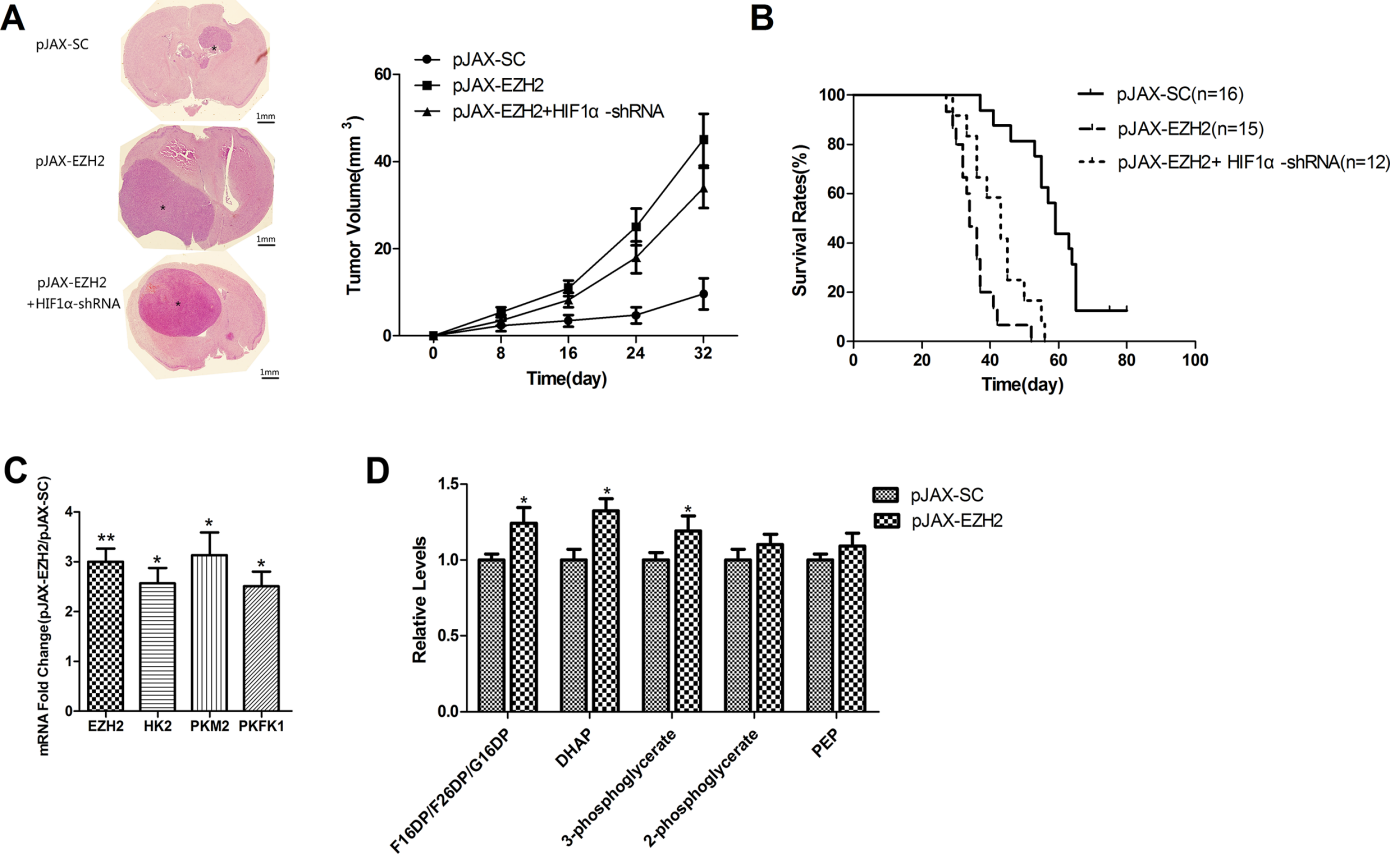
EZH2 is associated with to HIF1α-related metabolic reprogramming in intracranial glioma xenografts (**A**) Representative micrographs of H&E staining sections of mouse brain 32 days after intracranial implantation of U251 cells transfected with pJAX-SC, pJAX-EZH2 or pJAX-EZH2+HIF1α-shRNA. Asterisks indicate tumors (scale bar, 1 mm). The line chart shows the estimated tumor volumes at the indicated times (Nsc = 20, N_EZH2_ = 21, N_EZH2+ HIF1α-shRNA_= 20 ). (**B**) Curves showing the survival rates of the engrafted mice (Nsc = 16, N_EZH2_ = 15, N_EZH2+ HIF1α-shRNA_=12 ). (**C**) Levels of EZH2, HK2, PKM2 and PFK1 mRNA in the xenograft tumors. Data are presented as the mean ± s.d. from three independent experiments. *P < 0.05, **P < 0.01. (**D**) Levels of glycolytic intermediates. Data are presented as the mean ± s.d. from three independent experiments. *P < 0.05.

After implanting U251 cells transfected with pJAX-EZH2 or control vector into the brains of immunocompromised mice, we observed that up-regulation of EZH2 promoted tumor formation (Figure 3A) and decreased the survival of tumor-bearing mice (Figure 3B).

To investigate the effect of EZH2 on metabolic switching *in vivo*, we used qRT-PCR to assess the expression of HK2, PKM2, and PFK1 mRNA in xenograft tumors. The results showed that levels of all three enzymes were significantly increased in tumors derived from cells overexpressing EZH2 (Figure 3C), which suggests EZH2 overexpression leads to increases in glycolysis *in vivo.*

To further confirm the role of EZH2 in the glioblastoma metabolic switch *in vivo*, we used liquid chromatography-mass spectrometry (LC-MS) to examine the effect of EZH2 upregulation on levels of glycolytic metabolites. We found that within tumors overexpressing EZH2, levels of glycolytic intermediates were elevated (Figure 3D), whereas TCA cycle metabolites were only modestly changed (Supplementary Figure 2A). Thus overexpression of EZH2 is associated with a metabolic profile exhibiting a greater inclination towards glycolytic metabolism than the controls.

### EZH2 requires HIF1α to mediate glioblastoma metabolic adaptation

HIF1α is transcriptional factor known to activate metabolism-related genes such as HK2, GLUT-1 and PDK1 [26]. Given that a large number of EZH2 targets in the regulation of metabolism and tumorigenesis, we hypothesized that the actions of EZH2 in glioblastomas may be related to HIF1α.

**Figure 4:**
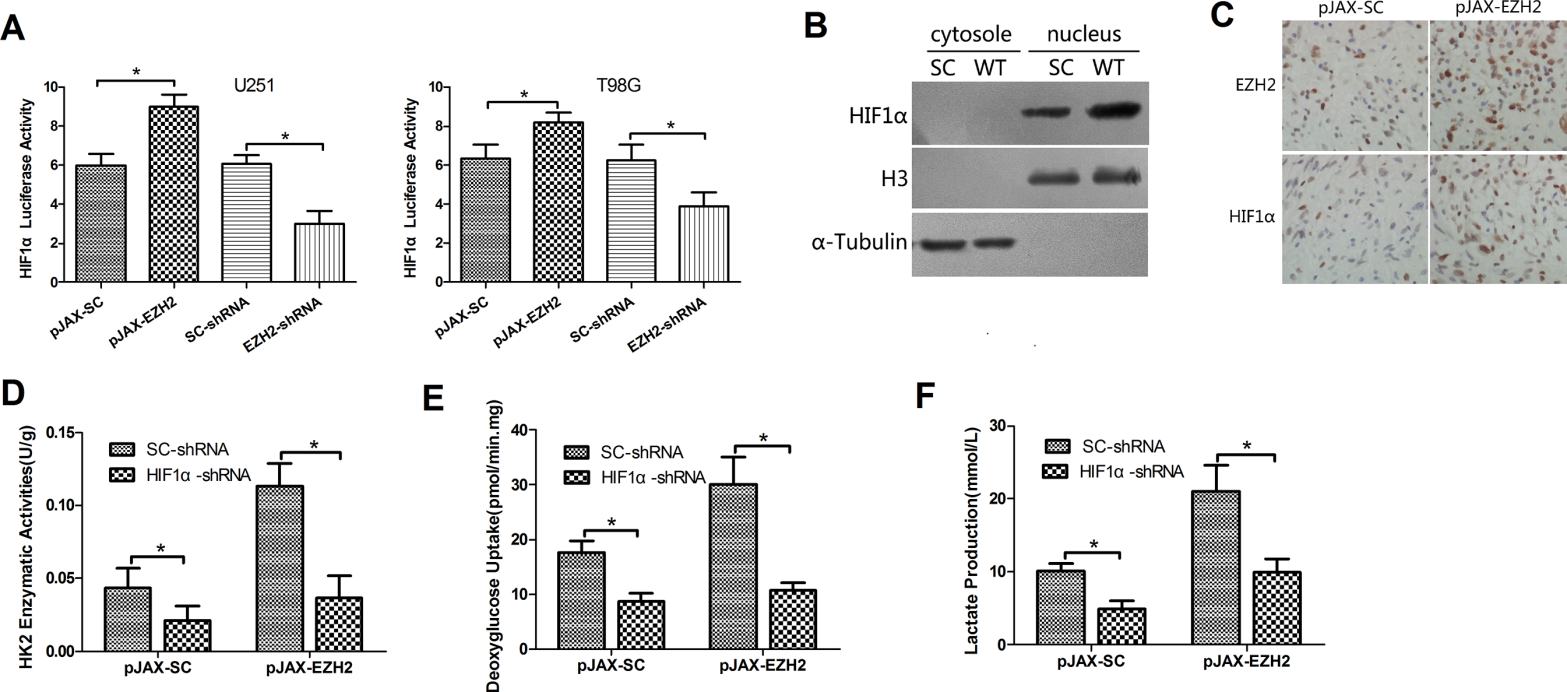
EZH2 requires HIF1α to mediate glioblastoma metabolic adaptation (**A**) U251 and T98G cells were transfected with pGL2-HIF1α; a vector-encoding EZH2-shRNA or SC-shRNA, or a vector-encoding wild-type EZH2 or its comparative control; and pSV-Renilla. Values in graphs are the mean of FLuc:RLuc activity ± s.d. from triplicate measurements. *P < 0.05. (**B**) Levels of HIF1α, histone H3 and α-tubulin proteins in the cytosolic and nuclear lysates from the control (SC) or EZH2-overexpressing (WT) U251 cells. (**C**) IHC was performed to detect HIF1α expression in xenograft tumors arising from control or EZH2-overexpressing glioblastoma cells. (**D**) Expression of HIF1α was silenced or not in U251 cells transfected with pJAX-SC or pJAX-EZH2. HK2 enzymatic activity was measured in the indicated cells. Data are presented as the mean ± s.d. from three independent experiments. *P < 0.05. Deoxyglucose uptake (**E**) and lactate production (**F**) were measured in the same sets of U251 cells. Data are presented as the mean ± s.d. from three independent experiments. *P < 0.05.

To test this hypothesis, we first performed luciferase reporter assay. Results showed that EZH2 overexpression increased HIF1α transcriptional activity and that EZH2 knockdown had the opposite effect (Figure 4A). In addition, overexpression of EZH2 increased HIF1α protein levels (Supplementary Figure 2B) under normoxic conditions. Moreover, in a nuclear/cytosol fractionation analysis, we detected increased levels of HIF1α protein in isolated nuclei from cells overexpressing EZH2 (Figure 4B).

To assess the effect of EZH2 on HIF1α *in vivo*, we first performed IHC with xenograft tumors derived from EZH2-overexpressing and control cells. EZH2 was increased in both the cytoplasm and nuclei cells overexpressing EZH2. Increaed HIF1α can be detected in EZH2 up-regulated cells (Figure 4C). Immunoblotting xenograft tumor tissues yielded similar results (Supplementary Figure 2C). Thus EZH2 appears to activate HIF1α *in vivo.*

Moreover, HIF1α knockdown suppressed enzymatic activities of HK2, PKM2 and PFK1 (Figure 4D, Supplementary Figure 2D, data not shown) in both control and EZH2-overexpressing cells, and reduced deoxyglucose uptake (Figure 4E, Supplementary Figure 2E) and lactate production (Figure 4F, Supplementary Figure 2F). And we co-transfected a HIF1α-shRNA vector into U251 cells along with pJAX-EZH2 vector. Knocking down HIF1α expression using shRNA partially inhibited tumor formation in mice (Figure 3A) and increased the survival of mice bearing tumors (Figure 3B). These results indicate that EZH2 requires HIF1α to mediate glioblastoma metabolic adaptation.

### EAF2 is an EZH2 target gene involved in the regulation of HIF1α

The serial analysis of gene expression (SAGE) database showed that EAF2 may be a target gene suppressed by EZH2. Previous studies have demonstrated that EAF2 binds to and stabilizes pVHL, a tumor inhibitor mediating HIF1α degradation [25]. In addition, EAF2 suppressed HIF1α activity by disrupting p300 recruitment and protected cells from hypoxia-induced cell death [27]. We therefore speculate that EAF2 may be a crucial linker connecting EZH2 up-regulation with HIF1α activation.

**Figure 5:**
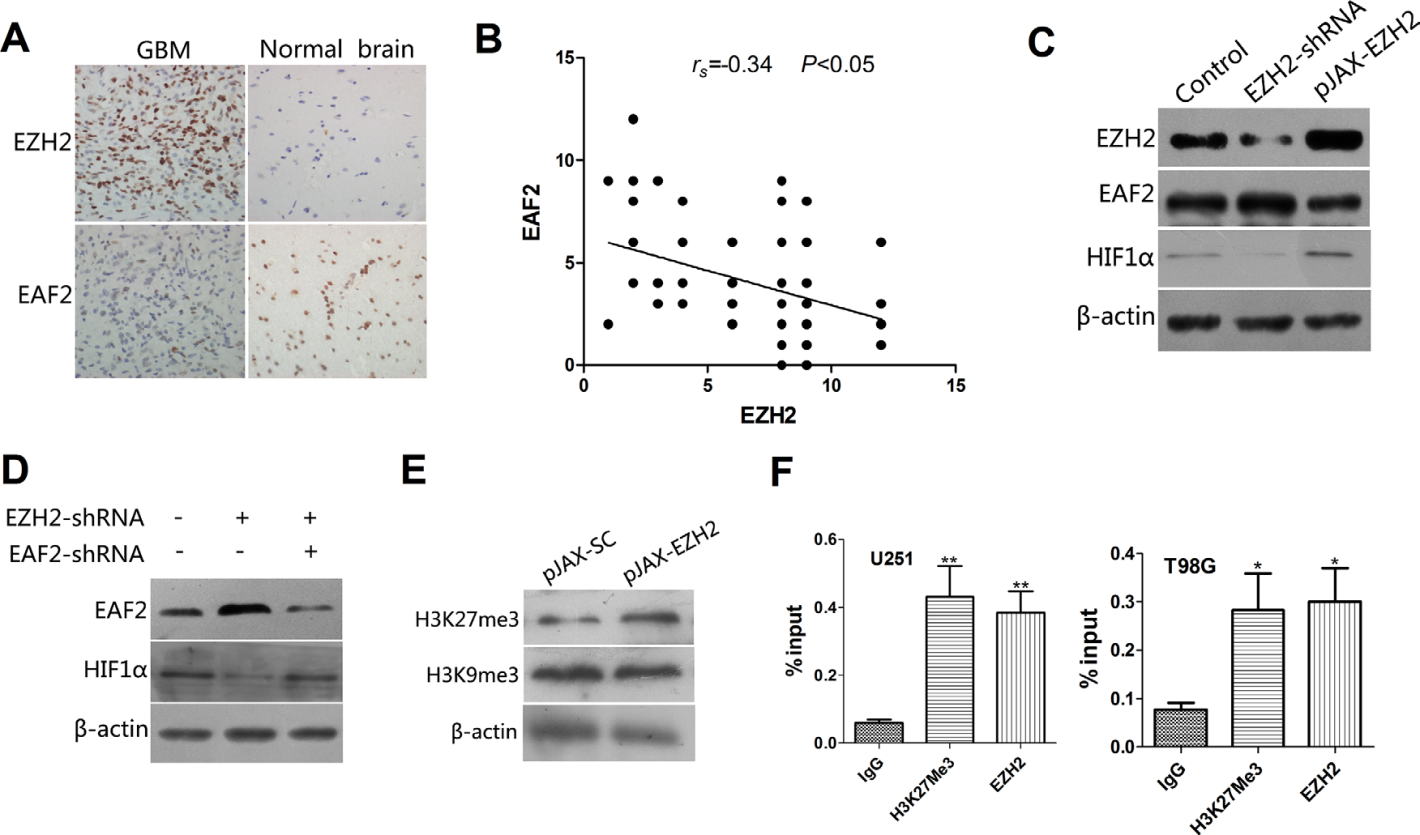
EAF2 is an EZH2 target gene involved in the regulation of HIF1α (**A**) IHC analysis of EZH2 and EAF2 expression in glioblastoma specimens and samples of normal brain tissue (400×). (**B**) Correlation between expression of EZH2 and EAF2 in 105 blioblastoma specimens (*P* < 0.05). (**C**) Levels of EAF2 and HIF1α protein were measured by immunoblotting U251 cells transfected with control vector, EZH2-shRNA or pJAX-EZH2. β-actin was used as a loading control. (**D**) EAF2 expression was silenced or not in U251 cells transfected with SC-shRNA or EZH2-shRNA. Immunoblots showing EAF2 and HIF1α levels in the indicated cells. β-actin was used as a loading control. (**E**) Immunoblots showing H3K27me3 and H3K9me3 levels in control and EZH2-overexpressing U251 cells. β-actin was used as a loading control. (**F**) U251 and T98G cells were subjected to ChIP analysis using antibodies against H3K27me3 or EZH2 with primers targeted to the promoter region of EAF2. Isotype matched IgG was used as a negative control. Data are presented as the mean ± s.d. from three independent experiments. *P < 0.05, **P < 0.01.

To test that idea, we first assessed EZH2 expression in 105 specimens of glioblastoma tissue and 12 samples of normal human brain tissue. IHC analysis showed that EZH2 was up-regulated in 81 (77%) of the glioblastoma specimens as compared to 2 (17%) of the normal brain tissue samples (*P* < 0.01). In addition, EAF2 was significantly downregulated in 72 (69%) glioblastoma samples as compared to 3 (25%) normal brain tissue samples (*P* < 0.01) (Figure 5A). There was thus a significant inverse correlation between expression of EZH2 and EAF2 (*p* < 0.05), suggesting EAF2 a potential target of EZH2 (Figure 5B).

When we then measured EAF2 levels in U251 (Figure 5C) and T98G (Supplementary Figure 3A) cells transfected with a plasmid encoding wild type EZH2 or shRNA targeting EZH2, we found that overexpression of EZH2 increased expression of HIF1α and decreased expression of EAF2. Not surprisingly, depletion of EZH2 had the opposite results. In addition, when cells were transfected with shRNAs targeting both EZH2 and EAF2, the downregulation of HIF1α due to EZH2 suppression was partially reversed by EAF2 depletion (Figure 5D, Supplementary Figure 3B). These data indicate that loss of EAF2 is required for EZH2-induced HIF1α expression.

Because EZH2 mainly acts to suppress gene expression through its methyl-transferase activity at Lys27 of histone H3, we examined levels of H3K27me3 in EZH2-overexpressing cells. The results showed that H3K27me3 was significantly increased by upregulated EZH2 expression. In comparison, H3K9me3 levels were nearly unchanged (Figure 5E, Supplementary Figure 3C). In addition, ChIP assays showed that EZH2 directly binds to the EAF2 promoter in U251 and T98G cells, and the H3K27me3 epigenetic mark was present in the EAF2 genomic locus (Figure 5F).

## DISCUSSION

Otto Warburg first showed in the 1920s that cancer cells prefer to carry out glycolysis even in the presence of sufficiently available oxygen. This is called the Warburg effect, and it promotes tumorigenesis [28, 29]. EZH2 is a well-known oncogene that is frequently upregulated in human cancers and is predictive of a poor prognosis [9, 10]. However, the precise mechanisms involved in EZH2-mediated tumorigenesis are far from clear. In the present study, we assessed the ability of EZH2 to modulate metabolic pathways. Our findings provide new evidence as to how EZH2 enhances tumorigenesis.

EZH2 is the catalytic subunit of polycomb repressive complex 2 (PRC2). The first clue of a role for polycomb proteins in mitochondrial metabolism came from a publication by Liu et al. in 2009 [30], who identified Bmi1, another key PRC subunit. Several years later, Zhang et al. reported that EZH2 and MICU1 were required to maintain mitochondrial membrane potential stability, and that they regulated tumor growth by modulating a mitochondria-dependent cell-death pathway [31]. On the other hand, the role of EZH2 in mitochondrial respiration and intracellular energetics has never been documented. Here, we found that oxygen consumption rates are reduced in glioblastoma cells depleted of EZH2, which suggests a deficiency in the TCA cycle. In addition, although overexpression of EZH2 exerted only a minor effect on mitochondrial oxidative capacity, glycolytic metabolism indicated by cellular deoxyglucose uptake and the activities of key enzymes involved in glycolysis and lactate production was significantly increased. These results suggest that EZH2 plays a crucial role in the regulation of the Warburg effect in glioblastomas.

Within tumors, oncogenes such as PI3K/Akt, c-Myc and HIF-1 regulate metabolic reprogramming [2, 5]. HIF1α is usually induced in a hypoxic environment. In the present study, however, exogenous overexpression of EZH2 increased HIF1α expression under normoxia. HIF1α regulation usually depends on oxygen-dependent protein stability. Under normoxic conditions, HIF1α is hydroxylated by a family of oxygen-dependent prolyl hydroxylases (PHD1-3), enabling pVHL to bind to and target HIF1α for ubiquitination and proteasomal degradation. In addition to hypoxia, various stimuli that affect post-translational regulation of HIF1α can also change HIF1α levels. For instance, somatic mutation of the gene encoding the tumor suppressor pVHL can promote progression of sporadic clear cell renal carcinoma. Individuals with this gene mutation also tend to suffer tumors in other parts of the body, including pheochromocytoma, retinal angioma, pancreatic cysts, and CNS hemangioblastoma [32, 33]. Overexpression of pVHL, on the other hand, inhibits the growth of gliomas [34].

A SAGE database showed that the tumor suppressor protein EAF2 is a likely target whose expression is repressed by EZH2, as depletion of EZH2 correlated with induction of EAF2. Indeed, we observed a significant inverse correlation between the expression of EZH2 and EAF2, further suggesting EAF2 is a target of EZH2. EAF2 binds to and stabilizes pVHL, thereby disrupting the HIF1α-mediated hypoxia signaling pathway [25]. Other groups also reported that EAF2 suppresses HIF1α activity by disrupting its interaction with coactivator CBP/p300 [27]. We therefore speculate that EAF2 is a negative regulator of HIF1α activity. Consistent with that idea, we showed that loss of EAF2 leads to upregulated expression of HIF1α. We also observed that EZH2 directly binds to the EAF2 promoter and suppresses its expression by adding an epigenetic methylation mark at H3K27. Another novel finding in this study is that EAF2 is dramatically downregulated in glioblastoma specimens, as compared to normal brain tissue.

In sum, our results demonstrate that EZH2 promotes tumorigenesis and malignant progression in part by activating glycolysis through an EAF2-HIF1α signaling axis. This finding may serve as the basis for new ideas about glioblastoma treatment.

## MATERIALS AND METHODS

### Cell lines and tissue samples

The U251 and T98G glioblastoma cell lines were obtained from Cell Bank of Type Culture Collection of Chinese Academy of Sciences (Shanghai, China). The Cells were maintained in Dulbecco's modified Eagle's medium (Life Technologies, Carlsbad, CA, USA) supplemented with 10% fetal bovine serum (HyClone, Logan, UT, USA) at 37°C with 5% CO_2_. A total of 105 glioblastoma tissue samples and 12 samples of normal human brain tissue were obtained from the Department of Neurosurgery at Provincial Hospital affiliated with Shandong University. The study was approved by Shandong University Ethics Committee, and all patients provided written informed consent according to the committee's regulations.

### Plasmids, shRNA

Knockdowns using small hairpin RNA (shRNA) were performed using pSuper.neo (OligoEngine, Seattle, WA, USA) following the manufacturer's instructions. The sequences were as follows: for EZH2, 5′-GAAATCTTAAACCAAGAAT-3′; for HIF1α, 5′-GAG CTTGCTCATCAGTTGC-3′; for EAF2, 5′-GAGTTGAA GGAAGCAGTAA-3′; and for scramble, 5′-GCGCGAT CCTAGACATGTG-3′. Plasmids encoding the shRNAs were transfected into T98G and U251 cells using Nanofectamine 2000 (Invitrogen, New York, NY, USA).

### Western blot analysis

Total proteins were extracted using lysis buffer containing 10 mmol/L Tris HCl (pH 7.4), 1% Triton X-100 and protease/phosphates inhibitors (Roche Diagnostics, Indianapolis, IN, USA), separated by 10% SDS-PAGE gel electrophoresis, transferred to PVDF membranes and probed with primary antibodies. The membranes were subsequently probed with horseradish peroxidase-conjugated secondary antibodies followed by development using an enhanced chemiluminescence detection system (Pierce, Rockford, IL, USA). The following primary antibodies were used: anti-EZH2 (Cell Signaling and Technologies, Boston, MA, USA); anti-HIF1α (Novus Biological Sciences, Littleton, CO, USA); anti-H3 (Abcam, Cambridge, MA, USA); anti-α-tubulin (Santa Cruz Biotechnology, Santa Cruz, CA, USA); anti-EAF2 (Abcam, Cambridge, MA, USA); anti-H3K27me3 (Cell Signaling and Technologies, Boston, MA, USA); anti-H3K9me3 (Abcam, Cambridge, MA, USA ) and anti-β-actin (Sigma-Aldrich, St Louis, Missouri, USA).

### Deoxyglucose uptake

Deoxyglucose uptake was assessed by measuring intracellular 2-[^3^H]deoxyglucose and phosphorylated deoxyglucose levels. Transfected cells were incubated for 10 min in medium containing 0.1 mM 2-deoxyglucose and 10 nM 2-[^3^H]deoxyglucose. The cells were then washed with cold PBS and lysed by the addition of 10 mM NaOH containing 0.1% Triton X-100. Lysates were subjected to liquid scintillation counting to determine ^3^H levels. The total protein concentration was used for normalization [2].

### Quantitative real-time PCR (qRT-PCR)

Total RNA from different cell lines was extracted using Trizol reagent (Gibco, Birmingham, MI, USA). Real-time PCR was performed using an ABI 7300 Fast Real-time PCR System (Applied Biosystems, Carlsbad, California, USA) with a SYBR Green PCR kit (Applied TaKaRa, Japan). The primer sequences were as follows: for EZH2, 5′-TTGTTGGCGGAAGCGTGTAAAATC-3′ (forward) and 5′-TCCCTAGTCCCGCGCAATGAGC-3′ (reverse); for HK2, 5′-TCCGTAGTGGGAAAAAG AGAA-3′ (forward) and 5′-GACAATGTGATCAA ACAGCTC-3′ (reverse); for PFK1, 5′-GGTGTACAAG CTTCTAGCTC-3′ (forward) and 5′-CAAGTTTAGA GCCACCTTGG-3′ (reverse); for PKM2, 5′-CCACTTGC TGTGCCAAATGGA-3′ (reverse) and 5′-GAAGGACTTT ACCTTCCAGGA-3′ (reverse); and for β-actin, 5′-CGCGA GAAGATGCCCAGATC-3′ (forward) and 5′-TCACC GGAGTCCATCACGA-3′ (reverse).

### HK2, PKM2, PFK1 enzymatic activities

The enzymatic activities of HK2, PKM2, PFK1 were measured in cell lysates using the respective enzyme activity assays (Jiemei Genetech. Corporation. Ltd., Shanghai, China) with the manufacturer's recommended protocols [2].

### Lactate production

Lactate levels in the culture medium were measured using a lactate assay kit (Nanjing Jiancheng Corporation Ltd, Shanghai, China) 24 h after the cells were seeded. The lactate assay is based on the reduction of tetrazolium salt 2-(4-iodophenyl)-3-(4-nitrophenyl)-5-phenyltetrazolium chloride to formazan in a nicotinamide adenine dinucleotide-coupled enzymatic reaction. Formazan is water-soluble and exhibits an absorption maximum at 492 nm. Total viable cell number was used for normalization [2].

### Oxygen consumption and extracellular acidification rates (OCR and ECAR)

OCR and ECAR were determined using a Seahorse XF24 extracellular Flux analyzer as previously described [35]. Briefly, cells were seeded into 24-well XF24 cell culture plates and allowed to grow for 24 h. Thirty minutes prior to the run, culture medium was replaced with running buffer, and plates were incubated for 30 min at 37°C for pH and temperature stabilization. The mitochondrial inhibitor, oligomycin (1 mM) and uncoupler FCCP (300 mM) were then applied. After all the measurements were completed, cells were dissociated and counted.

### Cell viability assay

Transduced cells were seeded into 96-well tissue culture plates to a concentration of 1,000 cells/well and incubated in medium with or without supplemented 4500 mg/l glucose. At the indicated time points, MTT was added to each well to a final concentration of 150 μg/ml. After incubation for 4 h at 37°C, absorbance at 570 nm was measured using a spectrophotometer (Benchmark, BIORAD, Hercules, CA, USA).

### Metabolite profiling

Metabolites were extracted in ice-cold methanol. Endogenous metabolite profiles were obtained using two liquid chromatography-tandem mass spectrometry (LC-MS) methods as described [36]. Data were acquired using a 4000 QTRAP mass spectrometer (Applied Biosystems/MDS Sciex, Nutley, NJ, USA). Multi-Quant software (Applied Biosystems/MDS Sciex, Nutley, NJ, USA) was used for analysis. Metabolite levels were normalized to protein content [37].

### *In vivo* tumor formation

Glioblastoma cells were intracranially injected into nude mice in accordance with a protocol approved by the Shandong University Institutional Animal Care and Use Committee and national regulatory standards. Briefly, 10^5^ transduced cells were injected into the right frontal lobes of athymic BALB/c nu/nu mice. At indicated times thereafter, the brains of the euthanized and PBS perfused mice were collected, fixed in 4% PFA, paraffin embedded, sectioned and stained with H&E using standard histological techniques. Tumor volumes were then determined by first measuring the maximal and minimal diameters of the tumor in the H&E-stained sections, after which volumes were calculated using the formula, tumor volume = (maximum diameter) × (minimum diameter)^2^ × 0.5 [38, 39].

### Luciferase reporter assay

Cells were plated in 48-well plates, transfected with the reporter plasmid pGL2-HIF1αLuc and control reporter pSV-Renilla together with shRNA-EZH2 or pJAX-EZH2 expression vector. FLuc and RLuc activities were determined using a dual-luciferase assay system (Promega, Madison, WI, USA) over a period of 24 h [2].

### Subcellular fractionation

Cells were lysed in hypotonic buffer using a Dounce homogenizer (40 strokes). Intact cells were removed by centrifugation at 53 × *g* for 10 min. After a second centrifugation at 800 × *g* for 10 min, the nuclei were collected as the pellet, washed, and lysed by sonication in isotonic buffer. The supernatant was collected as the cytosolic fraction [2].

### Immunohistochemistry (IHC)

Specimens were fixed in formalin and paraffin embedded. Five-μm-thick sections were prepared, and IHC staining was performed using antibodies against EZH2 (Cell Signaling and Technologies, Boston, MA, USA), HIF1α (Novus Biological Sciences, Littleton, CO, USA) and EAF2 (Abcam, Cambridge, MA, USA). The staining scores were determined as the percentage of positive cells multiplied by the intensity of positive staining on the slide (0, no expression or no positive staining; 1, 1–10% positive cells or light yellow; 2, 11–50% positive cells or brownish yellow; 3, 51–75% positive cells or brown; 4, > 75% positive cells or dark brown).

### Chromatin immunoprecipitation (ChIP)

U251 cells were cross-linked with 1% PFA and quenched by adding 125 mM glycine. Chromatin was isolated by adding cell lysis buffer (1% SDS, 10 mM EDTA, 50 mM Tris-HCl, pH 8.1, 1 mM PMSF) and sheared to fragments of 300–500 bp by sonication. Lysates were pre-cleared for 1–2 h using Salmon Sperm DNA/Protein A Agarose (EMD Millipore, Billerica, MA, USA), after which precipitation was induced using anti-H3K27me3 (Cell Signaling and Technologies, Boston, MA, USA) or anti-EZH2 (Cell Signaling and Technologies, Boston, MA, USA). An isotype matched IgG was used as a negative control. To then reverse the DNA cross-linking, the precipitates were incubated with pronase for 2 h at 42°C and 68°C for 8 h. The EAF2 promoter DNA in the precipitates was detected by qRT-PCR as described above using primers 5′- GCAGAAGGTGAGGGAGT-3′ (forward) and 5′-AAGATGGGAGAAGTGTTG-3′ (reverse).

### Statistical analysis

Quantitative data were expressed as the mean ± standard deviation (s.d.). Significance was tested using one-way ANOVA or Student's *t* test as appropriate. The Spearman rank correlation analysis was used to analyze the correlation of EZH2 and EAF2 expression. For *in vivo* studies, Kaplan Meier curves and log-rank analysis were performed using MedCalc software (Mariakerke, Belgium). Values of *P* < 0.05 were considered significant.

## Supplementary Materials


