# Supplementary material for: EZH2 promotes metabolic reprogramming in glioblastomas through epigenetic repression of EAF2-HIF1α signaling
Source: Oncotarget. 2016 Jun 1;7(29):45134–43. doi: 10.18632/oncotarget.9761 (PMC5216711; doi:10.18632/oncotarget.9761)
Supplement: Supplementary file 1 [file oncotarget-07-45134-s001.pdf]

EZH2 promotes metabolic reprogramming in glioblastomas through epigenetic repression of EAF2-HIF1α signaling

Supplementary Materials

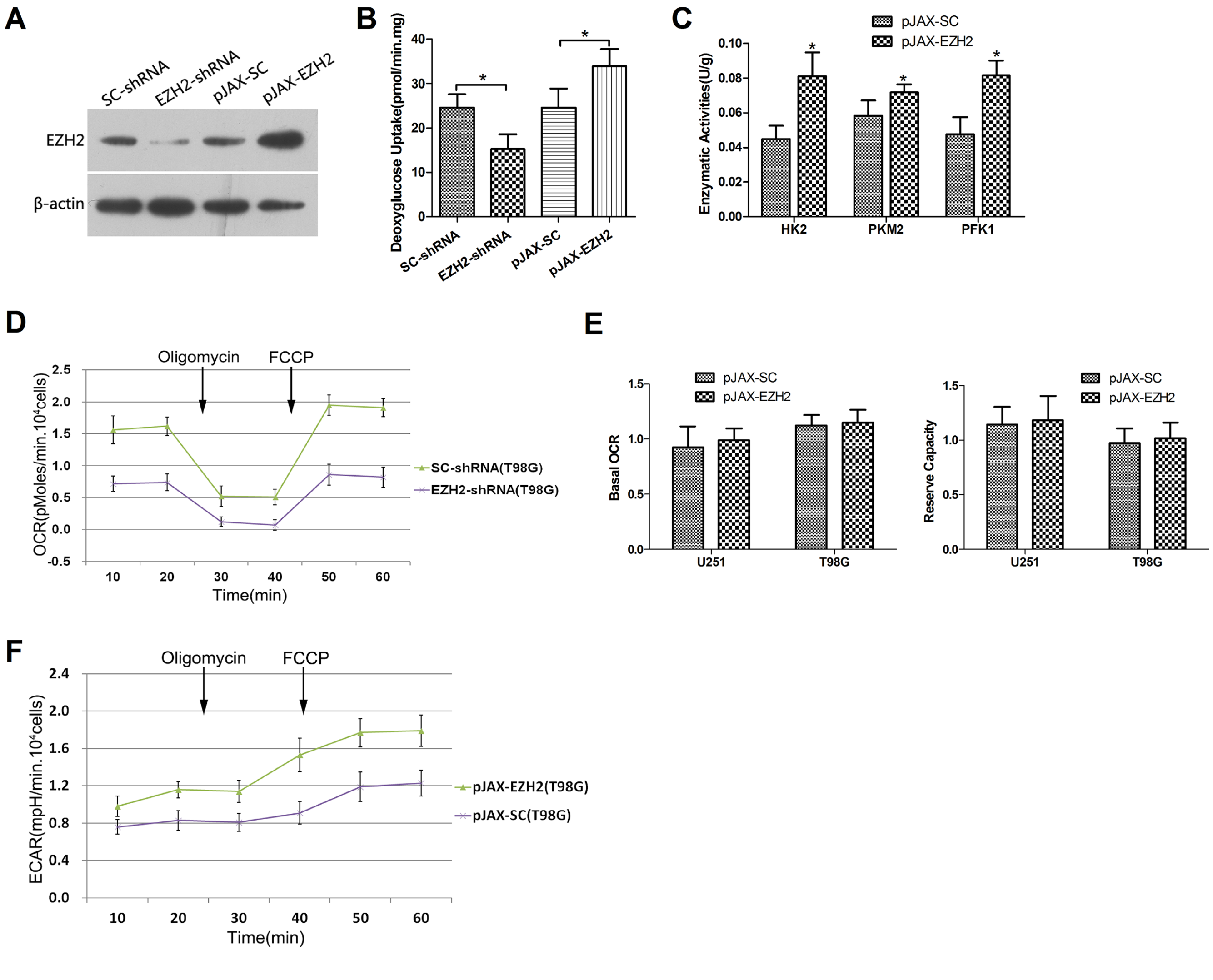

Supplementary Figure S1: Upregulation of EZH2 promotes glycolytic metabolism of glioblastoma cells. (**A**) Levels of EZH2 protein were analyzed by immunoblotting T98G glioblastoma cells transfected with SC-shRNA, EZH2-shRNA, pJAX-SC or pJAX-EZH2. β-actin served as a loading control. (**B**) Relative deoxyglucose uptake was measured in T98G cells transfected with SC-shRNA, EZH2-shRNA, pJAX-SC or pJAX-EZH2. Each bar represents the mean ± s.d. from three independent experiments. *$P < 0.05$. (**C**) Enzymatic activities of HK2, PKM2 and PFK1 were determined in the indicated T98G cells. Each bar represents the mean ± s.d. from three independent experiments. *$P < 0.05$. (**D**) OCRs were determined using a Seahorse XF24 analyzer with T98G glioblastoma cells transfected with SC-shRNA or EZH2-shRNA. Oligomycin (1 mM) and FCCP (300 mM) were administered as indicated. (**E**) Basal OCR and Reserve Capacity were calculated in the U251 and T98G glioblastoma cells transduced with pJAX-SC or pJAX-EZH2. Data are presented as mean± s.d. from three independent experiments. (**F**) ECARs were determined using a Seahorse XF24 analyzer with T98G glioblastoma cells transfected with pJAX-SC or pJAX-EZH2. Oligomycin (1 mM) and FCCP (300 mM) were administered as indicated.

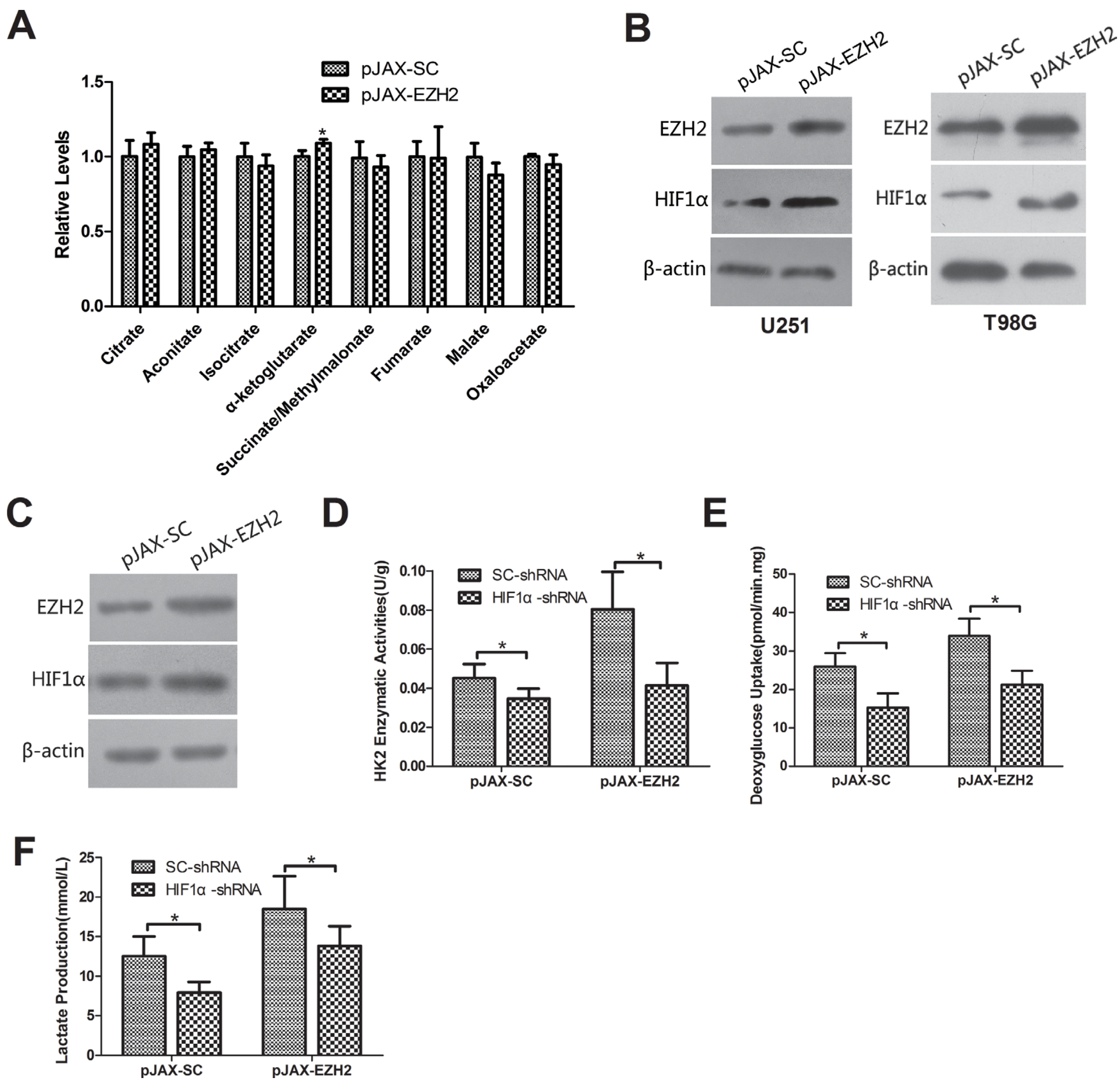

Supplementary Figure S2: Involvement of HIF1α activation in EZH2 mediated glycolytic metabolism. (**A**) Levels of TCA cycle intermediates were measured in the xenograft tumors. Each bar represents mean ± s.d. in triplicate. **P* < 0.05. (**B**) Immunoblot analysis was performed to determine the protein levels of HIF1α with cultured transduced cells. β-actin was used as a loading control. (**C**) Immunoblots showing EZH2 and HIF1α levels in xenograft tumors derived from the control and EZH2 up-regulated cells. β-actin was used as a loading control. (**D**) Expression of HIF1α was silenced or not in T98G cells transfected with pJAX-SC or pJAX-EZH2. HK2 enzymatic activity was measured in the indicated cells. Data are presented as the mean ± s.d. from three independent experiments. **P* < 0.05. Deoxyglucose uptake (**E**) and lactate production (**F**) were measured in the same sets of T98G cells. Data are presented as the mean ± s.d. from three independent experiments. **P* < 0.05.

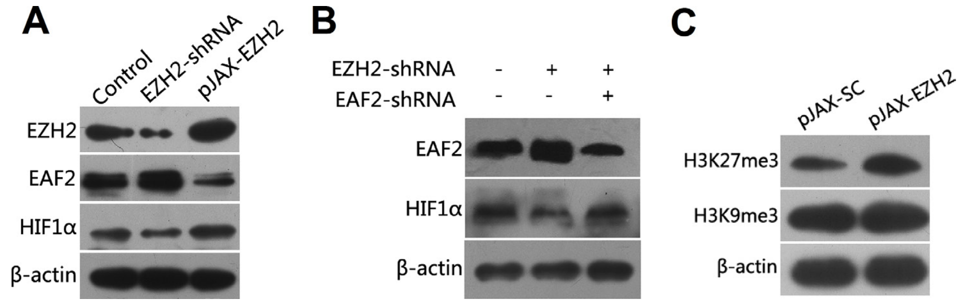

Supplementary Figure S3: EAF2 is involved in the regulation of HIF1α by EZH2. (**A**) Levels of EAF2 and HIF1α protein were measured by immunoblotting T98G cells transfected with control vector, EZH2-shRNA or pJAX-EZH2. β-actin was used as a loading control. (**B**) EAF2 expression was silenced or not in T98G cells transfected with SC-shRNA or EZH2-shRNA. Immunoblots showing EAF2 and HIF1α levels in the indicated cells. β-actin was used as a loading control. (**C**) Immunoblots showing H3K27me3 and H3K9me3 levels in control and EZH2-overexpressing T98G cells. β-actin was used as a loading control.
